# A new charge-tagged proline-based organocatalyst for mechanistic studies using electrospray mass spectrometry

**DOI:** 10.3762/bjoc.10.211

**Published:** 2014-08-28

**Authors:** J Alexander Willms, Rita Beel, Martin L Schmidt, Christian Mundt, Marianne Engeser

**Affiliations:** 1 University of Bonn, Kekulé-Institute of Organic Chemistry and Biochemistry, Gerhard-Domagk-Str. 1, D-53121 Bonn, Germany

**Keywords:** charge tag, electrospray, mass spectrometry, organocatalysis, proline, template

## Abstract

A new 4-hydroxy-L-proline derivative with a charged 1-ethylpyridinium-4-phenoxy substituent has been synthesized with the aim of facilitating mechanistic studies of proline-catalyzed reactions by ESI mass spectrometry. The charged residue ensures a strongly enhanced ESI response compared to neutral unmodified proline. The connection by a rigid linker fixes the position of the charge tag far away from the catalytic center in order to avoid unwanted interactions. The use of a charged catalyst leads to significantly enhanced ESI signal abundances for every catalyst-derived species which are the ones of highest interest present in a reacting solution. The new charged proline catalyst has been tested in the direct asymmetric inverse aldol reaction between aldehydes and diethyl ketomalonate. Two intermediates in accordance with the List–Houk mechanism for enamine catalysis have been detected and characterized by gas-phase fragmentation. In addition, their temporal evolution has been followed using a microreactor continuous-flow technique.

## Introduction

Electrospray ionization (ESI) mass spectrometry [1] has not only developed into a standard characterization method for an extremely broad variety of substances [2], but has also been recognized as a valuable tool for studying reaction mechanisms by transferring species of a reacting solution directly into the gas phase of a mass spectrometer [3–7]. The technique allows glimpses into the reacting solution as a function of time [8] and beyond that a characterization of transient intermediates by tandem mass spectrometry. ESI mass-spectrometric mechanistic studies have been reported for a broad range of reaction types ranging from transition metal-catalyzed polymerization [6,9] and coupling reactions [8,10–17] to purely organic Diels–Alder reactions [18–19] to cite only a few representative examples.

However, the detection of transient reactive species is often hindered by their very low concentration. A reacting solution of a catalytic transformation typically contains quite a number of different species. Side products, off-cycle resting states, reagent degradation products and impurities of various origins may be present in much higher concentration than the interesting reactive intermediates. Thus, ESI spectra of reacting solutions can be frustratingly complicated and the transient species of interest might be superposed with a large number of more intense background signals [20]. In quantification using ESI, the detection limit has been lowered significantly by selected ion monitoring in MS/MS mode [2]. Similarly, transient reactive species have been successfully extracted from the chemical noise by collision-induced dissociation (CID) MS/MS [20]. However, it is not possible to identify unknown or unexpected species by this strategy.

As a major drawback of ESI mass spectrometry in general, the signal intensity does not directly parallel the concentration, but the so called ESI response, i.e., the ionization probability during the ESI process [2,21]. Hence it happens that the reaction intermediates of interest are concealed by easily ionizable other compounds present in the reacting solution. A convenient approach to solve this problem is the use of covalently attached charge tags [6,8–9,12,22–23]. Charge-tagging the catalyst selectively enhances the signal abundances of all catalyst-derived species in a reacting solution and thus facilitates the identification of low-concentrated transient catalytic species. As a complementary approach, charge-tagged substrates have been used to easily identify (“fish for”) efficient catalysts [6,9].

Since the year 2000, enantioselective catalysis based on small organic metal-free molecules has become an enormously growing research topic [24–30]. A large variety of organocatalyzed reactions with high efficiency and selectivity are nowadays known so that organocatalysis complements current catalytic fields such as organometallic or enzymatic catalysis as an independent subdomain [24–30]. Parallel to the enormous growth of organocatalytic applications in synthesis, mechanistic studies on organocatalytic reactions [31–38] using ESI mass spectrometry [20,39–49] have been reported. The pioneering studies of List and Barbas [50] revealed that the amino acid L-proline is an effective catalyst for a great variety of organic reactions, such as the direct asymmetric aldol reaction, one of the most important C–C bond-forming reactions in organic synthesis [51]. The currently accepted mechanism suggests a central enamine intermediate which forms a Zimmerman–Traxler-like transition state with the acceptor substrate [36–37]. The activity and enantioselectivity achieved by proline in many cases is thought to be due to a templating effect of the OH group directing the aldehyde in a preferred position via hydrogen bonding [24–25]. It is still controversial whether oxazolidinone formation plays a pivotal role in the catalytic cycle or just serves as an rate limiting parasitic off-cycle equilibrium [31,33,35,52].

**Figure 1:**
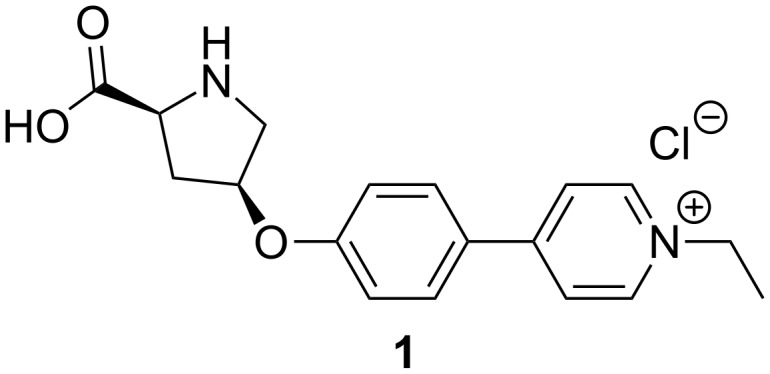
The new charge-tagged proline-derived catalyst **1**.

Thus, we aimed to synthesize a charge-tagged L-proline-based organocatalyst for mechanistic studies by ESIMS. Few proline derivatives carrying a covalently fixed charge have been reported by now [43,53]. They consist of an imidazolium salt attached to hydroxyproline via an ester group at the end of a flexible alkyl spacer. Interestingly, such charge tags can cause an enhancement of the catalytic performance through electrosteric activation [53], but backfolding can also alter and disturb the catalytic process and induce the formation of side products [43]. In order to fix the charge far away from the catalytic center and thus leave the original catalytic activity of L-proline preferably undisturbed, we chose a stiff 1-ethylpyridinium unit as charge-carrier separated from the catalytic center by a rigid phenyl linker (Figure 1).

**Scheme 1:**
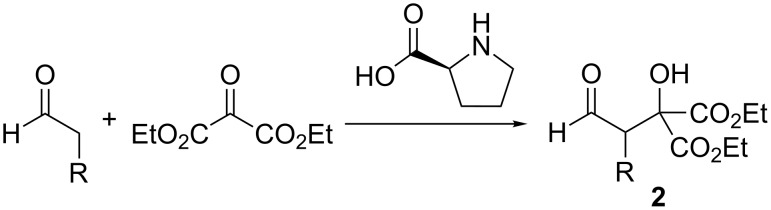
Inverse aldol reaction with aldehyde donors according to Jørgensen [54]. We studied the reaction for R = Ph (labelled **a** throughout this manuscript) and for R = Et (**b**).

We then tested the applicability of **1** for ESIMS mechanistic studies on the first “inverse” crossed aldol reaction (Scheme 1) published in 2002 by Jørgensen and coworkers [54] in which the aldehyde acts as the donor in contrast to the “normal” crossed aldol mechanism. It represents an interesting version of a typical proline-catalyzed reaction for which, to the best of our knowledge, mechanistic studies have not been reported so far.

## Results and Discussion

### Synthesis

**Scheme 2:**
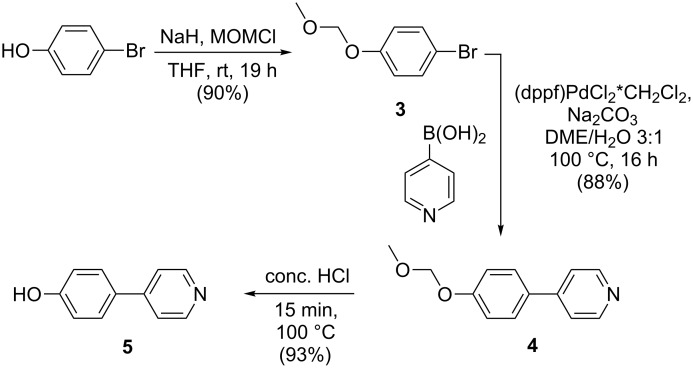
Synthesis of 4-(pyridin-4-yl)phenol (**5**).

Formation of the charge-carrying unit was accomplished starting from commercially available 4-bromophenol using a strategy reported by Diemer et al. [55]. Protection of the hydroxy group to yield **3** [56] was followed by Suzuki cross-coupling with commercial pyridine-4-boronic acid leading to **4**. Subsequent deprotection led to 4-(pyridine-4-yl)phenol (**5**) (Scheme 2) [55].

**Scheme 3:**
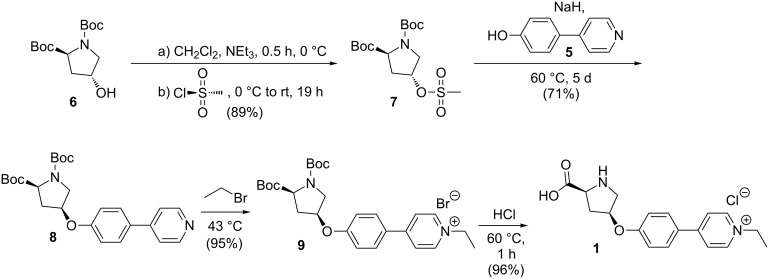
Synthesis of the charge-tagged proline catalyst **1**.

**Figure 2:**
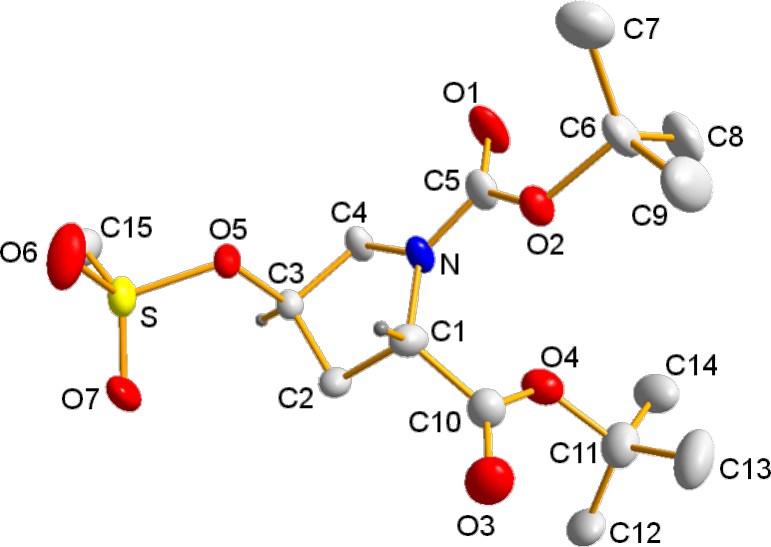
Molecular structure of **7** in the solid state.

The preparation of the charge-tagged catalyst **1** starting from doubly-protected hydroxyproline **6** [57] is depicted in Scheme 3. To introduce a suitable leaving group for the following step of the synthesis, compound **6** was mesylated to give the derivative **7** [58] for which crystals suitable for X-ray analysis have been obtained (Figure 2). An S_N_2 reaction with **5** [55] led to **8**. We abandoned our initial shorter synthetic route based on a Mitsunobu reaction leading from **6** directly to **8** due to severe purification difficulties. Compound **8** could be charge-tagged to **9** using ethyl bromide. Finally, the free catalyst **1** was obtained by acidic deprotection [59].

### Mechanism of the Jørgensen inversed aldol reaction

**Scheme 4:**
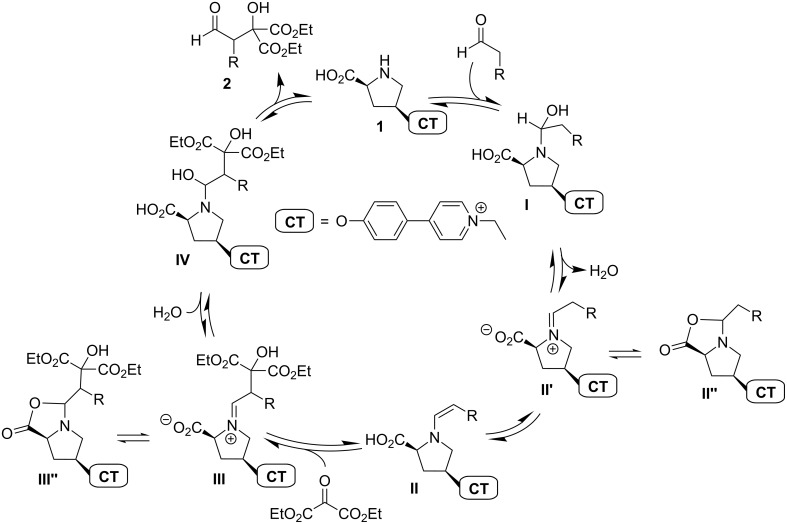
Proposed catalytic cycle [36–38] for the aldol reaction with aldehyde donors [54]; CT = charge tag, **a**: R = Ph, **b**: R = Et.

According to the mechanistic model for enamine catalysis from List and Houk [36–38], the aldol reaction from Jørgensen should proceed via the catalytic cycle shown in Scheme 4. We began our experiments with a test whether the charge tag does not disturb the catalysis. Indeed, **1** can achieve the formation of aldol products **2a** and **2b**, respectively, under the reaction conditions given in the literature [54]. Performed in simultaneous parallel reaction batches, **1** provides just about the same yields as unmodified proline and byproducts were not observed. Further, it is important to note that the substrates do not show any reaction when no catalyst, be it charge-tagged or not, is present in the solution.

**Figure 3:**
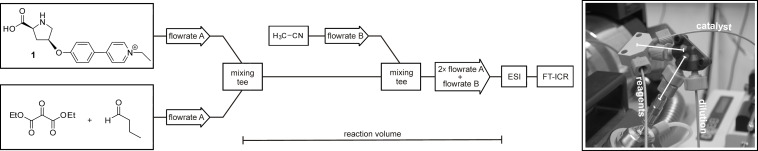
Experimental setup for continuous-flow ESIMS experiments using two mixing tee microreactors directly coupled to the ESI needle.

**Figure 4:**
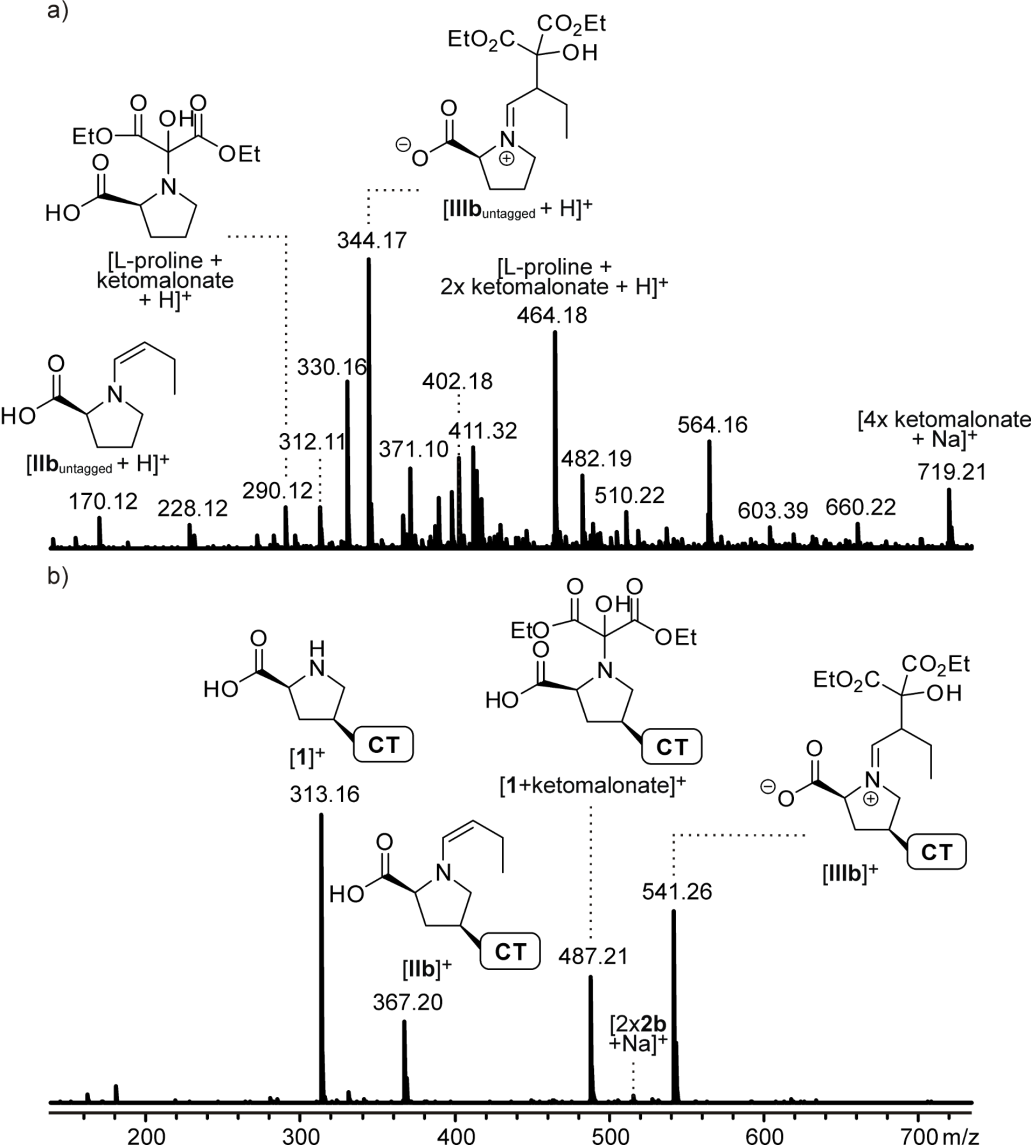
ESI mass spectra of acetonitrile solutions of diethyl ketomalonate and butyraldehyde (a) with unmodified L-proline or (b) with the charge-tagged catalyst **1** recorded with the continous-flow setup shown in Figure 3.

The easiest way of ESI reaction monitoring – mixing the reagents and measuring ESI spectra after various time intervals – is restricted to reaction times longer than approximately one minute and therefore not appropriate for fast conversions like aldol reactions. We thus decided to use a more complicated experimental setup of two mixing tees connected on-line to the mass spectrometer (Figure 3) to detect individual intermediates of both reactions by ESIMS. These so-called continuous-flow experiments [5,20] allow the sampling of reaction times down to seconds. Solutions of the reagents are mixed in the first microreactor and diluted to concentrations suitable for ESIMS in the second microreactor. The reaction time between the mixing event and the electrospray is determined by the flow rates and capillary lengths. Mass spectra of a solution of diethyl ketomalonate, butyraldehyde and unmodified L-proline or **1**, respectively, are depicted in Figure 4.

The mass spectra shown in Figure 4 do not exhibit abundant signals for the reactants, even though these are present in the solution in excess to all other species, a fact that is due to the poor ESI response of ketoesters and even more so of aldehydes. In the case of the proline-catalyzed solution (Figure 4a), the catalyst is not visible either, because of an instrumental discrimination of low masses unfortunately unavoidable with our instrument. Instead, two expected intermediates of the catalytic cycle indeed are observed in reasonable abundances (Figure 4a): The signal at *m*/*z* 170.12 corresponds to [**IIb**_untagged_ + H]^+^ and the one at *m*/*z* 344.17 is assigned to [**IIIb**_untagged_ + H]^+^. In contrast, signals for the remaining two intermediates **Ib**_untagged_ and **IVb**_untagged_ have not been found, which probably is due to their very low concentration in the reaction equilibria as well as to their facile fragmentation during ESI. Note that the group of Metzger has successfully achieved the detection of a similar intermediate for the aldol reaction beween acetone and selected benzaldehydes using rather unusual and presumably extremely soft ESI conditions, and their results indeed confirm its facile fragmentation [20].

Interestingly, the signal at *m*/*z* 290.12 can be assigned to a further transient species of the reaction – it corresponds to a protonated adduct of proline with diethyl ketomalonate. This adduct might simply be a non-covalently bound aggregate, i.e., a typical ESI phenomenon [2], but with regard to the rather low concentrations used here and the observation of the analogous species in the proton-free charge-tagged case (see below), we prefer its assignment to a hemiaminal species formed in analogy to intermediate **I** by interaction of the proline nitrogen with the keto group of the ketomalonate (structure depicted in Figure 4a). In this special case, elimination of water is not possible due to the lack of adjacent hydrogen atoms. Its formation thus represents an off-cycle equilibrium dead-end in the course of the intended inverse aldol reaction.

In light of the relatively high abundances observed for the reaction intermediates catalyzed by uncharged L-proline, the implementation of a charge tag might have been considered unneccessary. However, the effect of using the charge-tagged catalyst **1** is impressive (Figure 4b). The obvious reduction of spectral complexity and chemical noise due to the strongly enhanced ESI response of **1** and all its derivatives underlines the great benefits of the charge-tagging strategy. In addition to the catalyst **1** at *m*/*z* 313.16, the three transient species discussed above are found almost exclusively and in very high abundances, i.e., the enamine [**IIb**]^+^ at *m*/*z* 367.20, the iminium [**IIIb**]^+^ at *m*/*z* 541.26 and the side product [**1** + ketomalonate]^+^ at *m/z* 487.21. Please note that the abundance of the latter varies significantly between different reaction runs, in contrast to the signals of the other intermediates whose appearances are highly reproducible. Very similar findings are observed when phenylacetaldehyde instead of butyraldehyde is used. In particular, intermediates [**IIa**]^+^ and [**IIIa**]^+^ have been detected in high abundances. Again, we unfortunately have not been successful in finding suitable electrospray conditions to detect the fragile intermediates [**Ia**]^+^/[**Ib**]^+^ and [**IVa**]^+^/[**IVb**]^+^. More importantly, however, additional species that are not present in the unlabeled reference system have not been observed. There are no indications for an interference of the charge tag with the catalysis, in contrast to the findings with the flexible imidazolium-labeled proline derivatives reported previously [43].

**Figure 5:**
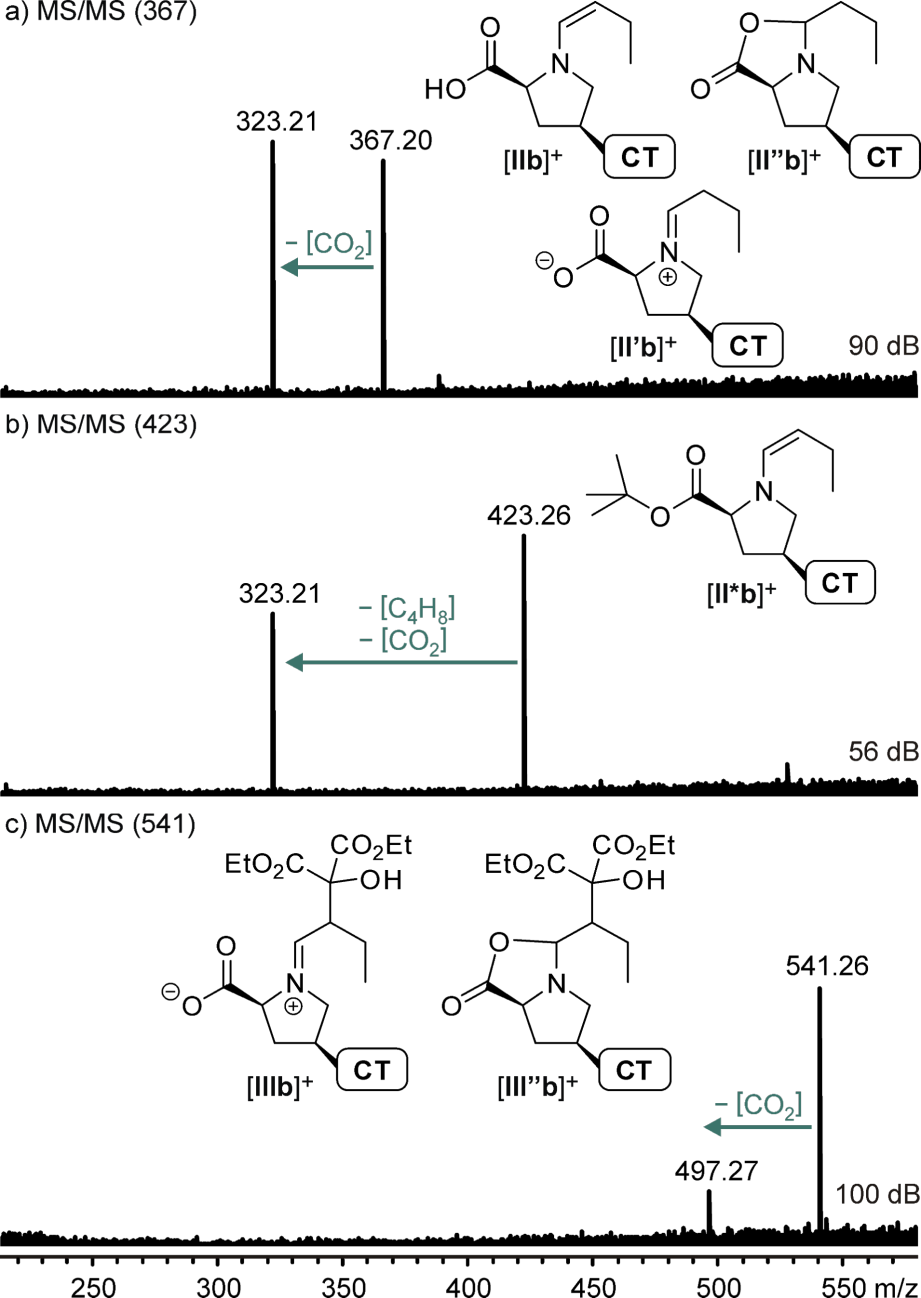
ESI(+) CID MS/MS spectra of mass-selected intermediates a) [**IIb**]^+^, b) the butyl ester derivative [II*b]^+^, c) [**IIIb**]^+^.

The mere detection of ions at *m*/*z* 415.20 (R = Ph) and 367.20 (R = Et), respectively, is not a proof for the presence of reactive enamines [**IIa**]**^+^**/[**IIb**]^+^ since the isomeric zwitterionic iminiums [**II’a**]^+^/[**II’b**]^+^ and oxazolidinones [**II’’a**]^+^/[**II’’b**]^+^ (Scheme 4) have the same elemental composition. Using one of the most important mass spectrometric means for structure elucidation, collision-induced dissociation (CID) experiments have been perfomed. The results for R = Et are shown in Figure 5.

All four mass-selected ions [**IIa**]^+^/[**IIb**]^+^ and [**IIIa**]^+^/[**IIIb**]^+^ show a very strong propensity to expel CO_2_ which even happens during the ESI process via in-source fragmentation when slightly harsher ionization conditions are used. This fragmentation is in perfect accordance with the zwitterionic iminium structures **II’** and **III** and can also be rationalized for the oxazolidinone alternative **II’’**, whereas it requires an additional hydrogen shift from the enamine structure **II**. On the other hand, the comparison with the fragmentation of the structurally related ion [II*b]^+^ (Figure 5b) is instructive. It doubtlessly possesses an enamine structure since it can neither form a zwitterionic iminium ion nor undergo lactonization to oxazolidinone **II’’b** because of its *tert*-butyl blocked carboxylic acid function. The fragmentation of [II*b]^+^ exclusively consists of a loss of [C_5_,H_8_,O_2_] which should correspond to a concerted or very fast stepwise elimination of isobutene and CO_2_ leading to the same product ion at *m*/*z* 323.21 as the expulsion of CO_2_ from *m*/*z* 367.20. Interpreting the isobutene loss as a closed-shell McLafferty-type rearrangement leads to the postulation of a (very short-lived undetected) intermediate enamine [**IIb**]^+^ which then obviously is able to undergo a facile CO_2_ elimination. Thus, the fragmentation spectra unfortunately do not allow a clear discrimination of the three possible structures **II**, **II’**, and **II’’**.

Marquez and Metzger mass-selected a signal corresponding to the protonated enamine from acetone and untagged L-proline and observed the elimination of CH_2_O_2_ (formic acid) instead of CO_2_ as main fragmentation component during CID [20]. The protonation during the ESI process presumably occurs at the nitrogen atom which enables a direct 1,2-elimination of formic acid. In our case, the respective charge-tagged species are detected in their original form without additional proton which explains the differing fragmentation route.

**Figure 6:**
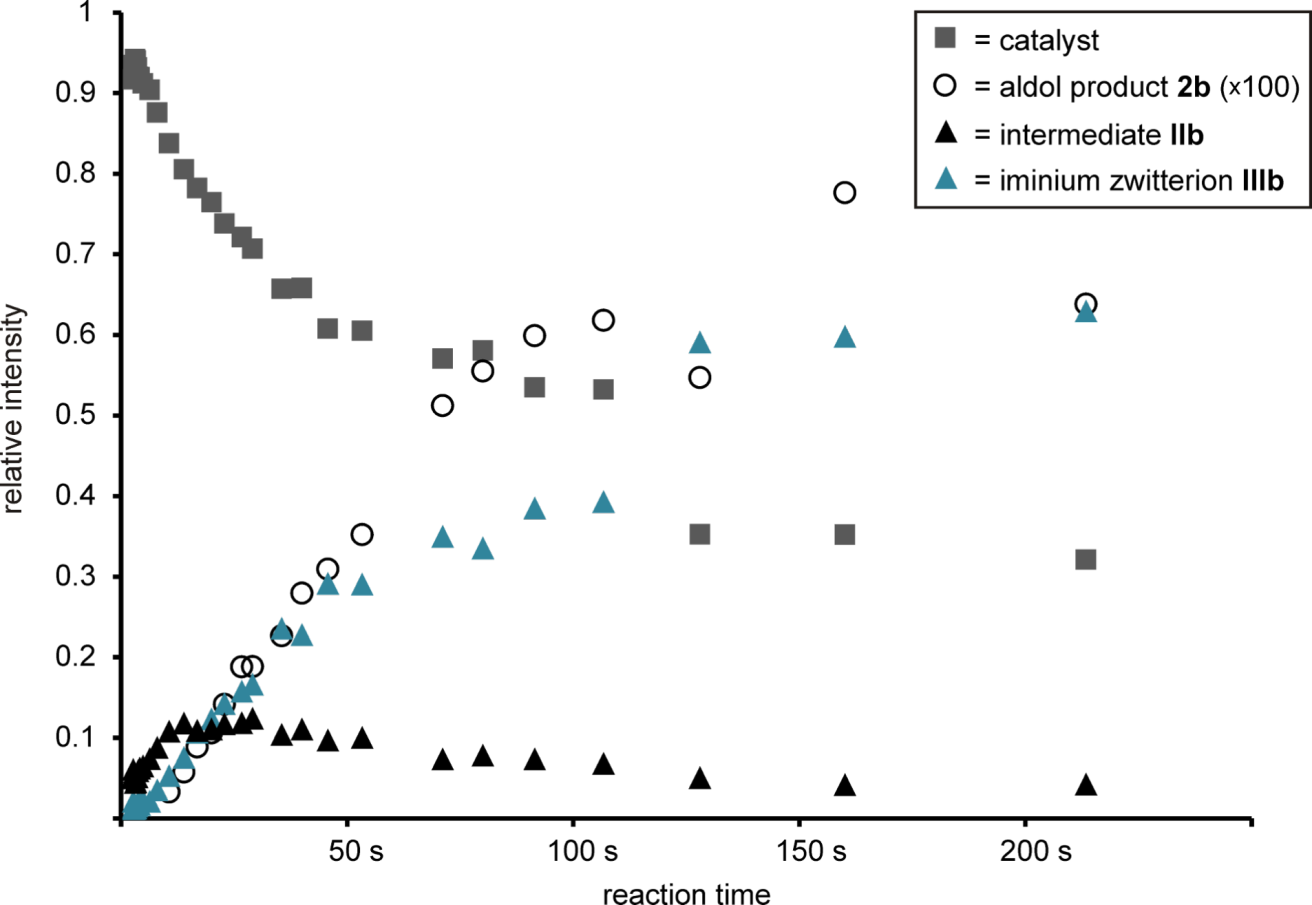
Normalized relative intensities in ESI spectra recorded for the inverse aldol reaction of butyraldehyde, diethyl ketomalonate and charge-tagged catalyst **1** at different time stages using the continous-flow setup with two microreactors shown in Figure 3.

To monitor the temporal evolution of intermediates during the aldol reaction with the continous-flow setup, series of ESI spectra at different reaction time stages have been recorded by varying the flow rate of the analyte solutions or by changing the length of the capillary connecting both mixing tees. A resulting graph of the normalized relative intensities vs calculated reaction time is provided in Figure 6.

The graph displays a rise of the concentration of **IIb** during the first seconds of the reaction accompanied by a decrease of **1**. Subsequently, an increase of **IIIb** occurs indicating that **IIIb** is formed out of **IIb** which is consistent with the mechanism in Scheme 4. The gradual increase of the final aldol product **2b** is visible as well; an enlargement of a factor of 100 has been used in the presentation of Figure 6 due to its much lower ESI response. Overall, these experiments reflect a very reasonable qualitative picture of the reaction behaviour and show that the use of catalyst **1** is suitable for the examination of L-proline-catalyzed reactions via ESIMS. However, we refrain from a quantitative kinetic modeling of the data to extract rate constants [8] because we encountered certain limitations of the method. Most importantly, we could not obtain an exact reproducibility of reaction times, probably because of quasi-unavoidable variations in the actual (dead) volumes of the setup inter alia due to varying minor capillary blockings. Moreover, we face a slight increase of signal abundances with measuring time (not reaction time) because analytes gradually accumulate in the system the longer their solutions are passed through. The extent of this effect depends on the height of the flow rate which necessarily has to be changed when observing a reaction process with the continuous-flow method. Nevertheless, we would like to emphasize that the quality of the experiments surely suffices to depict the “chronological trend” of the reaction process.

## Conclusion

We present the synthesis of the charge-tagged L-proline derived catalyst **1** in which a rigid phenylpyridine linker fixes the charge tag far away from the catalytically active center in order to avoid unwanted interactions. In a comparative continous-flow electrospray mass spectrometric study, the new charged catalyst **1** and neutral L-proline have been used to investigate the proline-catalyzed inverse crossed aldol reaction of aldehydes with diethyl ketomalonate. Two key intermediates of the List–Houk mechanism for enamine catalysis in addition to a transient off-cycle species could be observed experimentally. The use of **1** further allows facile access to a qualitative picture of the temporal evolution of catalyst-containing intermediates. We plan to use the new proline catalyst with a non-interfering charge-label presented here as a tool to study the templating role of the hydroxy group in L-proline-catalyzed reactions in the gas-phase in the near future.

## Experimental

### Synthesis

Reactions under inert gas atmosphere were performed under argon using standard Schlenk techniques and oven-dried glassware prior to use. Thin-layer chromatography was performed on aluminum TLC plates silica gel 60F_254_ from Merck. Detection was carried out under UV light (254 and 366 nm). Products were purified by column chromatography on silica gel 60 (40–63 µm) from Merck. The ^1^H and ^13^C NMR spectra were recorded on a Bruker Avance 300 spectrometer, at 300.1 and 75.5 MHz, or a Bruker AM 400, at 400.1 MHz and 100.6 MHz, at 293 K, respectively. The ^1^H NMR chemical shifts are reported on the δ scale (ppm) relative to residual non-deuterated solvent as the internal standard. The ^13^C NMR chemical shifts are reported on the δ scale (ppm) relative to deuterated solvent as the internal standard. Signals were assigned on the basis of ^1^H, ^13^C, HMQC, and HMBC NMR experiments. Most solvents were dried, distilled, and stored under argon according to standard procedures. 4-Bromophenol, 4-pyridinylboronic acid, *trans*-*N*-(*tert*-butoxycarbonyl)-4-hydroxy-L-proline, L-proline, diethyl ketomalonate, phenylacetaldehyde and butyraldehyde were used as received from commercial sources.

**4-Bromophenol methoxymethyl ether (3)** [56]: 4-Bromophenol (3.00 g, 17.2 mmol) was dried under reduced pressure and dissolved in dry THF (160 mL) under inert gas atmosphere. NaH (1.32 g, 52.0 mmol) was added and the mixture was stirred at rt for 0.5 h. Methoxymethyl chloride (2.08 mL, 26.0 mmol) was added dropwise and the resulting suspension was stirred for 19 h. The reaction was quenched by the addition of a MeOH/H_2_O mixture (1:1, 150 mL) and the aqueous phase was extracted with CH_2_Cl_2_ (4 × 50 mL). The combined organic extracts were dried with MgSO_4_ and the solvents were removed in vacuo. The crude product was purified by column chromatography on silica gel using cyclohexane/ethyl acetate (10:1) as eluent (*R*_f_ 0.61). Compound **3** was obtained as colorless oil (3.38 g, 90%). The spectroscopic data confirm the reported ones [56].

**4-[4-(Methoxymethoxy)phenyl]pyridine (4)** [55]: 4-Bromophenol methoxymethyl ether (**3**, 1.5 g, 6.9 mmol), 4-pyridinylboronic acid (1.02 g, 7.5 mmol), [1,1'-bis(diphenylphosphino)ferrocene]palladium(II) dichloride dichloromethane complex (0.22 g, 0.28 mmol) and Na_2_CO_3_ (8.78 g, 82.4 mmol) were suspended in a H_2_O/1,2-dimethoxyethane mixture (1:3, 75 mL), heated to 100 °C und stirred for 16 h. The resulting mixture was filtered and the filtrate was mixed with H_2_O (75 mL) and CH_2_Cl_2_ (75 mL) for phase separation. The aqueous phase was extracted with CH_2_Cl_2_ (2 × 75 mL), the combined organic extracts were dried with MgSO_4_ and the solvents were removed in vacuo. The crude product was purified by column chromatography on silica gel using ethyl acetate with 5% triethylamine as eluent (*R*_f_ 0.50). Compound **4** was obtained as white solid (1.31 g, 88%). The spectroscopic data confirm the reported ones [55].

**4-(Pyridine-4’-yl)phenol (5)** [55] and **(2*S*,4*R*)-*tert*-butyl *N*-*tert*-butyloxycarbonyl-4-hydroxyprolinate (6)** [57] were prepared according to literature protocols.

**(2*S*,4*R*)-*tert*-Butyl *N*-*tert*-butoxycarbonyl-4-oxymethanesulfonyloxyprolinate (7)** was prepared according to a known procedure [58] which was slightly modified: (2*S*,4*R*)-*tert*-butyl *N*-*tert*-butyloxycarbonyl-4-hydroxyprolinate (**6**, 3.14 g, 10.9 mmol) was dissolved in CH_2_Cl_2_ (55 mL) and cooled to 0 °C. Triethylamine (3.01 mL, 21.5 mmol) was added and the mixture was stirred for 0.5 h. Methanesulfonyl chloride (1.33 mL, 17.1 mmol) was added dropwise over 10 min and the resulting solution was stirred overnight without further cooling. The reaction was quenched by the addition of a saturated solution of NaHCO_3_ (70 mL) and the aqueous phase was extracted with CH_2_Cl_2_ (2 × 50 mL). The combined organic extracts were dried with MgSO_4_ and the solvents were removed in vacuo. The crude product was purified by column chromatography on silica gel using cyclohexane/ethyl acetate (2:3) as eluent (*R*_f_ 0.61). Compound **7** was obtained as colorless solid (3.55 g, 89%). Crystals suitable for X-ray analysis have been obtained by slow diffusion of cyclohexane into a Et_2_O solution of **7**. ^1^H NMR (400 MHz, CD_3_OD) δ 5.26 (s, 1H, CH_2_C*H*CH_2_), 4.30–4.23 (m, 1H, CH_2_C*H*N), 3.83–3.76 (m, 1H, NC*H_2_*CH), 3.70–3.63 (m, 1H, NC*H_2_*CH), 3.14 (s, 3H, S-C*H_3_*), 2.67–2.56 (m, 1H, CHC*H_2_*CH), 2.28–2.19 (m, 1H, CHC*H_2_*CH), 1.50–1.44 (m, 18H, C(C*H_3_*)_3_) ppm; ^1^H NMR (400 MHz, CD_3_OD, 333 K) δ 5.26 (s, 1H, CH_2_C*H*CH_2_), 4.30–4.26 (m, 1H, CH_2_C*H*N), 3.83–3.74 (m, 1H, NC*H_2_*CH), 3.72–3.63 (m, 1H, NC*H_2_*CH), 3.12 (s, 3H, S-C*H_3_*), 2.67–2.54 (m, 1H, CHC*H_2_*CH), 2.30–2.20 (m, 1H, CHC*H_2_*CH), 1.48 (s, 9H, C(C*H_3_*)_3_), 1.46 (s, 9H, C(C*H_3_*)_3_) ppm; ^13^C NMR (100 MHz, CD_3_OD) δ 173.0/172.9 (*C*═O), 155.6 (*C*═O), 83.1 (*C*(CH_3_)_3_), 82.1/81.9 (*C*(CH_3_)_3_), 80.6/79.9 (CH_2_*C*HCH_2_), 59.5/59.4 (CH_2_*C*HN), 54.1/53.7 (N*C*H_2_CH), 38.3 (S-*C*H_3_), 37.3 (CH*C*H_2_CH), 28.6 (C(*C*H_3_)_3_), 28.3/28.2 (C(*C*H_3_)_3_) ppm; ^13^C NMR (125 MHz, CD_3_OD, 333 K) δ 172.9 (*C*═O), 155.6 (*C*═O), 83.1 (*C*(CH_3_)_3_), 82.1 (*C*(CH_3_)_3_), 80.5/79.8 (CH_2_*C*HCH_2_), 59.5 (CH_2_*C*HN), 53.9/53.5 (N*C*H_2_CH), 38.5 (S-*C*H_3_), 38.3/37.3 (CH*C*H_2_CH), 28.7 (C(*C*H_3_)_3_), 28.3 (C(*C*H_3_)_3_) ppm; HRESIMS (*m*/*z*): [M + Na]^+^ calcd for C_15_H_27_NNaO_7_S, 388.1406; found, 388.1398. The NMR data are consistent with the reported ones measured in CDCl_3_ [58,60].

**(2*S*,4*S*)-*tert*-Butyl *N*-*tert*-butoxycarbonyl-4-(4-(pyridine-4-yl)phenoxy)prolinate (8):** 4-(Pyridine-4’-yl)phenol (**5**, 0.63 g, 3.7 mmol) and NaH (0.14 g, 5.5 mmol) were dissolved in dry DMSO (100 mL) under inert gas atmosphere. The suspension was heated to 60 °C and stirred for 1.5 h. (2*S*,4*R*)-*tert*-Butyl *N*-*tert*-butoxycarbonyl-4-methanesulfonyloxyprolinate (**7**, 1.33 g, 3.7 mmol) was added and the mixture was stirred for 5 d at 60 °C. The reaction progress was controlled by thin-layer chromatography. The reaction was quenched by the addition of H_2_O (100 mL) and the aqueous phase was extracted with CH_2_Cl_2_ (2 × 80 mL) and with Et_2_O (2 × 80 mL). The combined organic extracts were dried with Na_2_SO_4_ and the solvents were removed in vacuo. Remaining DMSO was removed by distillation. The crude product was purified by column chromatography on silica gel using cyclohexane/ethyl acetate (1:3) with 5% triethylamine as eluent (*R*_f_ 0.50). Compound **8** was obtained as colorless solid (1.14 g, 71%).^1^H NMR (300 MHz, CD_3_OD) δ 8.55–8.45 (m, 2H, *H*_o-py_), 7.72 (d, ^3^*J* = 8.8 Hz, 2H, *H*_m-ph_), 7.67 (d, ^3^*J* = 6.1 Hz, 2H, *H*_m-py_), 7.06/7.00 (d, ^3^*J* = 8.8 Hz, 2H, *H*_o-ph_), 5.06 (s, 1H, CH_2_C*H*CH_2_), 4.41–4.26 (m, 1H, CH_2_C*H*N), 3.86–3.72 (m, 1H, NC*H_2_*CH), 3.72–3.57 (m, 1H, NC*H_2_*CH), 2.67–2.49 (m, 1H, CHC*H_2_*CH), 2.47–2.37 (m, 1H, CHC*H_2_*CH), 1.51–1.40 (m, 18H, C(C*H_3_*)_3_) ppm; ^13^C NMR (75 MHz, CD_3_OD) δ 172.5/172.5 (*C*═O), 159.7/159.5 (*C*═O), 156.0 (*C*_ph_−O), 150.4 (*C*_o-py_−H), 150.1 (*C*_p-py_−C), 131.7/131.4 (*C*_p-ph_−C), 129.6/129.4 (*C*_m-ph_−H), 122.5 (*C*_m-py_−H), 117.3/117.2/117.2 (*C*_o-ph_−H), 82.9/82.7/82.6 (*C*(CH_3_)_3_), 81.9/81.6 (*C*(CH_3_)_3_), 77.2/76.2 (CH_2_*C*HCH_2_), 60.1/59.8 (CH_2_*C*HN), 53.6/53.2 (N*C*H_2_CH), 37.1/36.4 (CH*C*H_2_CH), 28.7/28.6 (C(*C*H_3_)_3_), 28.4 (C(*C*H_3_)_3_) ppm; HRESIMS (*m*/*z*): [M + H]^+^ calcd for C_25_H_33_N_2_O_5_, 441.2389; found, 441.2390.

**4-(4-((3*S*,5*S*)-1,5-Bis(*tert*-butoxycarbonyl)pyrrolidine-3-yloxy)phenyl**)**-1-ethylpyridinium bromide (9):** (2*S*,4*S*)-*tert*-Butyl *N*-*tert*-butoxycarbonyl-4-(4-(pyridine-4-yl)phenoxy)prolinate (**8**, 0.22 g, 0.5 mmol) was dissolved in bromoethane (13.0 mL, 174.2 mmol), heated to 43 °C and stirred for 5 d. Bromoethane was removed in vacuo. Compound **9** was obtained as yellowish-brown solid (0.26 g, 95%). ^1^H NMR (400 MHz, CD_3_OD) δ 8.89 (d, ^3^*J* = 6.8 Hz, 2H, *H*_o-py_), 8.35 (d, ^3^*J* = 6.8 Hz, 2H, *H*_m-py_), 8.08/7.99 (m, 2H, *H*_m-ph_), 7.18/7.11 (d, ^3^*J* = 8.9 Hz, 2H, *H*_o-ph_), 5.19–5.13 (m, 1H, CH_2_C*H*CH_2_), 4.63 (q, ^3^*J* = 7.4 Hz, 2H, C*H_2_*CH_3_), 4.43–4.36 (m, 1H, CH_2_C*H*N), 3.89–3.73 (m, 1H, NC*H_2_*CH), 3.71–3.58 (m, 1H, NC*H_2_*CH), 2.71–2.55 (m, 1H, CHC*H_2_*CH), 2.47–2.40 (m, 1H, CHC*H_2_*CH), 1.67 (t, ^3^*J* = 7.4 Hz, 3H, CH_2_C*H_3_*), 1.52-1.42 (m, 18H, C(C*H_3_*)_3_) ppm; ^13^C NMR (100 MHz, CD_3_OD) δ 172.5/172.4 (*C*═O), 162.0 (*C*_ph_−O), 157.0 (*C*_p-py_−C), 155.9 (*C*═O), 145.2 (*C*_o-py_−H), 131.2/131.1 (*C*_m-ph_-H), 127.5 (*C*_p-ph_-C), 125.0 (*C*_m-py_−H), 117.8/117.7 (*C*_o-ph_−H), 82.7/82.6 (*C*(CH_3_)_3_), 81.6 (*C*(CH_3_)_3_), 77.6/76.6 (CH_2_*C*HCH_2_), 60.0/59.8 (CH_2_*C*HN), 57.2 (*C*H_2_CH_3_), 53.6/53.2 (N*C*H_2_CH), 37.1/36.3 (CH*C*H_2_CH), 28.7/28.6 (C(*C*H_3_)_3_), 28.3 (C(*C*H_3_)_3_), 16.7 (CH_2_*C*H_3_) ppm; HRESIMS (*m*/*z*): [M]^+^ calcd for C_27_H_37_N_2_O_5_, 469.2697; found, 469.2692.

**4-(4-((3*S*,5*S*)-5-Carboxypyrrolidin-3-yloxy)phenyl)-1-ethylpyridinium bromide (1)** was prepared analogous to a known procedure [59] and obtained as beige-brown solid (96%). ^1^H NMR (400 MHz, CD_3_OD) δ 8.92 (d, ^3^*J* = 6.4 Hz, 2H, H_o-py_), 8.37 (d, ^3^*J* = 6.4 Hz, 2H, H_m-py_), 8.06 (d, ^3^*J* = 8.6 Hz, 2H, H_m-ph_), 7.27/7.20 (d, ^3^*J* = 8.6 Hz, 2H, H_o-ph_), 5.42 (s, 1H, CH_2_C*H*CH_2_), 4.73–4.58 (m, 3H, C*H_2_*CH_3_ and CH_2_C*H*N), 3.82–3.62 (m, 2H, NC*H_2_*CH), 2.85–2.73 (m, 1H, CHC*H_2_*CH), 2.71–2.63 (m, 1H, CHC*H_2_*CH), 1.67 (t, ^3^*J* = 7.3 Hz, 3H, CH_2_C*H_3_*) ppm; ^13^C NMR (100 MHz, CD_3_OD) δ 171.0 (*C*═O), 160.9 (*C*_ph_−O), 156.9 (*C*_p-py_−C), 145.4 (*C*_o-py_−H), 131.0 (*C*_m-ph_-H), 128.5 (*C*_p-ph_-C), 125.2 (*C*_m-py_−H), 118.0 (*C*_o-ph_−H), 77.3/76.7 (CH_2_*C*HCH_2_), 59.8 (CH_2_*C*HN), 57.4 (*C*H_2_CH_3_), 52.7 (N*C*H_2_CH), 35.7 (CH*C*H_2_CH), 16.7 (CH_2_*C*H_3_) ppm; HRESIMS (*m*/*z*): [M]^+^ calcd for C_18_H_21_N_2_O_3_, 313.1547; found, 313.1563.

**Diethyl 2-hydroxy-2-(2-oxo-1-phenylethyl)malonate (2a)** was prepared according to the reported procedure [54] and obtained as orange oil. The synthesis was carried out twice, once using L-proline (82%), once using **1** as catalyst (79%).

**Diethyl 2-hydroxy-2-(1-oxobutan-2-yl)malonate (2b)** was prepared according to the reported procedure [54] and obtained as orange oil (using L-proline: 83%, using **1:** 80%).

**Crystal structure determination:** X-ray crystallographic analysis of **7** was performed on a Nonius KappaCCD diffractometer using graphite monochromated Mo Kα radiation (λ = 0.71073 Å). Intensities were measured by fine-slicing φ- and ω-scans and corrected for background, polarization and Lorentz effects. A semi-empirical absorption correction was applied for the data sets following Blessing’s method [61]. The structure was solved by direct methods and refined anisotropically by the least squares procedure implemented in the ShelX program system [62]. The hydrogen atoms were included isotropically using the riding model on the carbon atoms. Selected data: Crystal dimensions 0.36 × 0.20 × 0.11 mm^3^, C_15_H_27_NO_7_S, *M* = 365.4424, orthorhombic, space group *P*2_1_2_1_2_1_, *a* = 7.44860(10), *b* = 8.94580(10), *c* = 28.7024(4) Å, α = 90°, β = 90°, γ = 90°, *V* = 1912.55(4) Å^3^, *Z* = 4, *ρ* = 1.269 g cm^−3^, μ = 0.203 mm^−1^, F(000) = 748, 18641 reflections (2θ_max_ = 27.99°) measured (4566 unique, *R*_int_ = 0.0606, completeness = 99.4%), *R* (*I* > 2σ(*I*)) = 0.0708, *wR*_2_ (all data) = 0.2054. GOF = 1.052 for 224 parameters and 14 restraints, largest diff. peak and hole 1.557/−0.438 e Å^3^. CCDC-1016532 contains the supplementary data for this structure. These data can be obtained free of charge from http://www.ccdc.cam.ac.uk/data_request/cif.

### Mass spectrometry

ESI mass spectra were recorded on a Bruker APEX IV Fourier-transform ion cyclotron resonance (FT–ICR) mass spectrometer with a 7.05 T magnet and an Apollo electrospray (ESI) ion source equipped with an off-axis 70° spray needle. Analyte solutions were fed into high pressure PEEK mixing tees from Alltech and then introduced into the ion source with a single- and a dual syringe pump from Cole Parmer and KD Scientific, respectively, at flow rates of 50 µL/h to 16 mL/h. The continuous-flow experiments were performed with a setup of two mixing tees. The first one was used for mixing a solution of both butyraldehyde and diethyl ketomalonate (each 2 mmol/L) with a solution of the catalyst (1 mmol/L) and the second mixing tee served for sufficient dilution. Different reaction times were achieved by changing either the length of the capillary connecting both tees or by varying the flow rate. The theoretical reaction time between **1** and the reactants has been calculated from the experimental flow rates considering the volumes of both mixing tees and the connecting capillaries and under the assumption that the dilution in the second mixing tee decreases the reaction rate in the fashion of a bimolecular elementary reaction. For longer reaction times (>200 s), the results can be compared with the ones from simple ESI measurements recorded at various times after offline mixing of the reaction partners. The results from both techniques match reasonably well, even though the reaction times calculated for the continous-flow setup seem to be slightly underestimated.

Ionization parameters were adjusted as follows: capillary voltage: −2.380 to −3.800 V; end plate voltage −2300 to 3320 V; capexit voltage: 50 to 100 V; skimmer voltages: 7 to 17 V; temperature of drying gas: 50 to 80 °C. Nitrogen was used as nebulizing (1.38 to 4.14 bar) and drying gas (1.38 to 3.10 bar). The ions were accumulated in the instruments hexapole for 0.3 to 0.9 s, introduced into the FT–ICR cell which was operated at pressures below 10^−10^ mbar, and detected by a standard excitation and detection sequence. Collision-induced fragmentation was performed by on-resonance excitation with argon gas pulsed into the ICR cell followed by a pumping delay of 3–5 s. For each measurement, 8 to 64 scans were averaged. All signal assignments are based on exact mass determinations.
